# Developing Embedded Taxonomy and Mining Patients’ Interests From Web-Based Physician Reviews: Mixed-Methods Approach

**DOI:** 10.2196/jmir.8868

**Published:** 2018-08-16

**Authors:** Jia Li, Minghui Liu, Xiaojun Li, Xuan Liu, Jingfang Liu

**Affiliations:** ^1^ School of Business East China University of Science and Technology Shanghai China; ^2^ Xi’an Research Institute of Hi-Tech Xi'an China; ^3^ School of Management Shanghai University Shanghai China

**Keywords:** labeled-LDA, physicians, topic modeling, topic taxonomy, Web-based review

## Abstract

**Background:**

Web-based physician reviews are invaluable gold mines that merit further investigation. Although many studies have explored the text information of physician reviews, very few have focused on developing a systematic topic taxonomy embedded in physician reviews. The first step toward mining physician reviews is to determine how the natural structure or dimensions is embedded in reviews. Therefore, it is relevant to develop the topic taxonomy rigorously and systematically.

**Objective:**

This study aims to develop a hierarchical topic taxonomy to uncover the latent structure of physician reviews and illustrate its application for mining patients’ interests based on the proposed taxonomy and algorithm.

**Methods:**

Data comprised 122,716 physician reviews, including reviews of 8501 doctors from a leading physician review website in China (haodf.com), collected between 2007 and 2015. Mixed methods, including a literature review, data-driven-based topic discovery, and human annotation were used to develop the physician review topic taxonomy.

**Results:**

The identified taxonomy included 3 domains or high-level categories and 9 subtopics or low-level categories. The physician-related domain included the categories of medical ethics, medical competence, communication skills, medical advice, and prescriptions. The patient-related domain included the categories of the patient profile, symptoms, diagnosis, and pathogenesis. The system-related domain included the categories of financing and operation process. The F-measure of the proposed classification algorithm reached 0.816 on average. Symptoms (Cohen *d*=1.58, Δ*u*=0.216, *t*=229.75, and *P*<.001) are more often mentioned by patients with acute diseases, whereas communication skills (Cohen *d*=−0.29, Δ*u*=−0.038, *t*=−42.01, and *P*<.001), financing (Cohen *d*=−0.68, Δ*u*=−0.098, *t*=−99.26, and *P*<.001), and diagnosis and pathogenesis (Cohen *d*=−0.55, Δ*u*=−0.078, *t*=−80.09, and *P*<.001) are more often mentioned by patients with chronic diseases. Patients with mild diseases were more interested in medical ethics (Cohen *d*=0.25, Δ*u* 0.039, *t*=8.33, and *P*<.001), operation process (Cohen *d*=0.57, Δ*u* 0.060, *t*=18.75, and *P*<.001), patient profile (Cohen *d*=1.19, Δ*u* 0.132, *t*=39.33, and *P*<.001), and symptoms (Cohen *d*=1.91, Δ*u*=0.274, *t*=62.82, and *P*<.001). Meanwhile, patients with serious diseases were more interested in medical competence (Cohen *d*=−0.99, Δ*u*=−0.165, *t*=−32.58, and *P*<.001), medical advice and prescription (Cohen *d*=−0.65, Δ*u*=−0.082, *t*=−21.45, and *P*<.001), financing (Cohen *d*=−0.26, Δ*u*=−0.018, *t*=−8.45, and *P*<.001), and diagnosis and pathogenesis (Cohen *d*=−1.55, Δ*u*=−0.229, *t*=−50.93, and *P*<.001).

**Conclusions:**

This mixed-methods approach, integrating literature reviews, data-driven topic discovery, and human annotation, is an effective and rigorous way to develop a physician review topic taxonomy. The proposed algorithm based on Labeled-Latent Dirichlet Allocation can achieve impressive classification results for mining patients’ interests. Furthermore, the mining results reveal marked differences in patients’ interests across different disease types, socioeconomic development levels, and hospital levels.

## Introduction

### Background

With the popularity of the internet, more and more people search Web-based information when they make decisions regarding health care providers. Among those sources, Web-based physician reviews are most frequently cited. Physician review websites (PRWs) permit patients and third-party reviewers to grade both physicians and hospitals in popular Web-based forums. Therefore, Web-based physician reviews reduce uncertainty surrounding the experience and serve as a valuable source of information for patients making choices [1-3]. Examples of PRWs include Healthgrades.com [4], Vitals.com [5], RateMDs.com [6], and a host of smaller, less-organized websites. Despite the subjectivity of evaluations in the reviews, Web-based reviews are an important source of Web-based information because they are perceived as more reliable and trustworthy than traditional information sources [7]. In addition, the Web-based physician review not only provides valuable information for patients who want to make a wise choice among health care providers but also provides some insights into physicians and hospitals who intend to improve their services in the future [1,8]. In summary, Web-based physician reviews are invaluable gold mines that merit further investigation [2,8,9].

### Literature Review

Most extant studies on physician reviews only use numeric variables, such as volume (number of reviews) and valence (rating score), in their empirical analysis and fail to consider the information in the review text; for example, Hao [10] examined the development of the Web-based doctor review practice in China, focusing on the number of doctors and specialty areas available for Web-based review, the number of Web-based reviews for these doctors, the specialty areas where doctors are more likely to be reviewed, and the quantitative rating score distribution. Li et al [11] examined how the proportion and position of negative reviews on such websites influence readers’ willingness to choose the reviewed physician and found that an increase in the proportion of negative reviews led to a reduced willingness to use the physician’s services. In addition, a primacy effect was found for negative reviews, such that readers were less willing to use the physician’s services when negative reviews were presented before the positive reviews. Yang et al [1] explored the effect of the patient- and system-generated information on patients’ Web-based searches, evaluations, and decisions, suggesting that the positive patient- and system-generated information on physicians’ service quality positively impacted patients’ reactions at different stages. Moreover, they found that synergies between the patient-generated and the system-generated information positively associated with patients’ decisions to consult a physician.

However, the information from numeric ratings is very limited compared with the whole review text, leading to a substantial loss of valuable information. The description of a medical consultation is multifaceted [12]. Therefore, a single number, such as a rating score of satisfaction or attitude, might not be adequate for patients to identify entire information relevant to physician choice. In addition, Web-based rating scores may not accurately capture or serve as a proxy for the physician quality. Recently, Daskivich et al [13] indicated that Web-based ratings of specialist physicians fail to predict objective measures of the quality of care or peer assessment of the clinical performance. Furthermore, Web-based ratings tend to be exaggerated at the upper or lower ends of the quality spectrum [14]. Therefore, recent studies on physician reviews focused more on the rich information embedded in the review text; for example, Hao and Zhang [9] automatically extracted hidden topics from Web-based physician reviews using text mining techniques to examine what Chinese patients said about their doctors and whether these topics differ across various specialties. Hao et al [15] compared the positive and negative reviews of obstetrics and gynecology doctors from the two most popular Web-based doctor rating websites in the United States and China. Grabner-Kräuter and Waiguny [2] explored how certain characteristics of physician reviews affected the evaluation of the review and users’ attitude toward the rated physician and suggested a positive main effect of the number of reviews as well as an interaction effect with the style of the review. If the physician received only a few reviews, fact-oriented reviews induced a more favorable attitude toward the physician compared with emotional reviews; however, there was no such effect when the physician received many reviews. Lockie et al [16] investigated which textual and content elements were related to the perceived usefulness of Web-based reviews for doctors (general practitioners) and indicated that reviews with a more narrative or experiential style were generally perceived as more useful than more fact-based or very short reviews.

The prior research most related to this study is Hao and Zhang [9], in which authors extracted hidden topics from Web-based physician reviews using the Latent Dirichlet Allocation (LDA). However, there are several differences between this study and that by Hao and Zhang [9]. First, in the study conducted by Hao and Zhang [9], only the data-driven approach was used to derive the topics. However, in this study, we use a mixed-methods approach consisting of a literature review, data-driven-based topic discovery, and human annotation approaches to rigorously and systematically develop a physician review taxonomy. Second, there is no theoretical basis in the study conducted by Hao and Zhang [9]. In this study, Maslow’s hierarchy of needs theory [17] is used to build a theoretical framework to guide the research questions and the whole paper.

Third, only LDA is used in the study conducted by Hao and Zhang [9]. In this study, both LDA and labeled-LDA are used. As an unsupervised machine-learning approach, LDA is used to find the initial topics embedded in the reviews, whereas labeled-LDA, as a semisupervized machine-learning approach, is used to classify the reviews into topics. Fourth, Hao and Zhang [9] made a comparison across 4 specialty areas (ie, internal medicine, surgery, obstetrics-pediatric, and Chinese medicine) as well as between Chinese doctor reviews and American doctor reviews. In this study, we focus on patients’ interests across different disease types and hospital levels.

### Research Questions

In this study, we are first interested in developing a systematic topic taxonomy embedded in the physician reviews. Although many studies have explored the text information of physician reviews, very few have focused on developing a systematic topic taxonomy embedded in the physician reviews. Web-based physician reviews are multifaceted in nature. Without such a topic taxonomy, the findings of the physician review text mining research are hard to compare directly. Hence, the first step toward mining physician reviews is to determine how the natural structure or dimension is embedded in reviews. Of note, extant studies that involve physician review topics are *ad hoc* in nature. There is a lack of research that develops the topic taxonomy in a rigorous and systematic manner. Therefore, the first research question is proposed as follows.:

RQ1: What topics are embedded within the Web-based physician review?

Second, patients’ interests and needs may not be universal across different disease types and hospital levels. According to Maslow’s hierarchy of needs theory [17], low-level needs, such as physiological requirements and safety, must be satisfied before high-level needs, such as self-fulfillment, are pursued. When a need is mostly satisfied, it no longer motivates, and the next higher need takes its place. Therefore, some topics that reflect low-level needs should be important for low-level hospitals, and some other topics that reflect high-level needs should be important for high-level hospitals. Hence, in this study, we are further interested in investigating different interests and needs of different patients. Therefore, the second research question is proposed as follows:

RQ2: Do different patients have different interests and needs? If so, how do their interests and needs differ across different disease types and hospital levels?

In summary, we aim to develop a hierarchical topic taxonomy to uncover the latent structure of physician reviews and illustrate its application for mining patients’ interests based on the proposed taxonomy and algorithm in this study.

### Data

In this study, we focus on a leading Web-based PRW, “Good Doctor” (haodf.com [18]), in China. China has its own hospital categorization system. According to the facilities and technique strength, Chinese hospitals are classified into three levels, with the A-level being the best and the C-level the worst. A-level hospitals have the best physicians and medical equipment; these not only provide specialized medical services but also undertake many teaching and research tasks. However, C-level hospitals focus on mass coverage and only provide basic medical services for community members.

The website haodf.com [18] was set up to help Chinese consumers to find good and appropriate specialists for their health care problems based on Web-based reviews [10]. As of July 2017, the platform had 507,365 registered doctors and 2,745,304 physician reviews. The physicians who received reviews on haodf.com [18] cover all major specialty areas. Because haodf.com [18] was designed to find good doctors, more physicians are from high-level hospitals (eg, A-level hospitals) than low-level hospitals (eg, C-level hospitals), especially from the largest and most famous hospitals in Beijing and Shanghai. Anyone who visited a physician can write a review on the website. The review process is anonymous because the website masks the reviewers’ username. Other personal information about reviewers is also unavailable to the public. In addition, reviewers can voluntarily disclose their sociodemographic information and health status. Similar to other health rating systems, such as healthgrades.com [4], users are allowed to rate a physician with scores and comments. Writing a review is voluntary. For more information about the development of Web-based physician reviews in China and haodf.com [18], please refer to the analysis of Hao [10].

A total of 122,716 physician reviews of 8501 doctors from haodf.com [18] were collected by a Web spider. The dataset covers the most frequently reviewed top 9 diseases (diabetes, gastric cancer, hypertension, hyperthyroidism, infantile diarrhea, infantile pneumonia, infertility, influenza, and liver cancer) from 2007 to 2015 on the website; this distribution is different from that of Western countries. For example, the incidence rates for liver and gastric cancer are higher in China than those in the United States [19]. Table 1 summarizes the descriptive statistics information for the review data.

**Table 1:** Descriptive statistics of the review data.

Disease	Reviews (N=122,716), n (%)	Doctors (N=10,764), n (%)	Reviews per physician	Average length per review (words)
Infertility	71,556 (58.31)	3573 (33.19)	20.0	444
Infantile pneumonia	21,839 (17.80)	2148 (19.96)	10.2	435
Infantile diarrhea	711 (0.60)	275 (2.60)	2.6	499
Influenza	1796 (1.46)	643 (6.00)	2.8	408
Hyperthyroidism	3028 (2.47)	872 (8.10)	3.5	383
Diabetes	20,849 (16.99)	2627 (24.41)	7.9	360
Liver cancer	1679 (1.37)	288 (2.70)	5.8	569
Gastric cancer	1053 (0.86)	231 (2.20)	4.6	560
Hypertension	205 (0.20)	107 (1.00)	1.9	369

## Methods

### Framework

**Figure 1:**
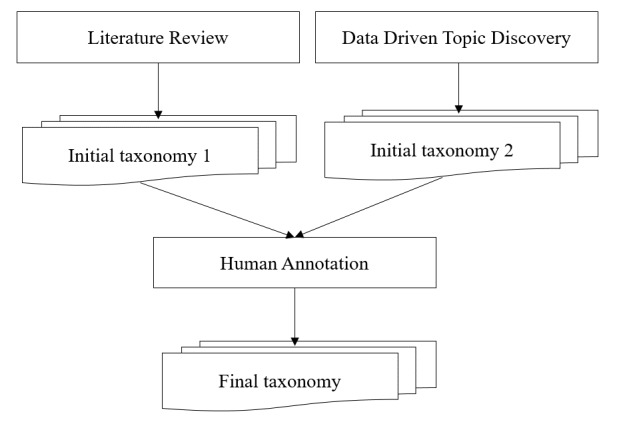
Framework for the development of the physician review topic taxonomy.

Figure 1 shows the proposed methodology to discover the hidden topics and build the taxonomy. The framework consists of three major steps as follows: summarizing topics from the literature; discovering hidden topics using a data-driven approach; and finalizing the topic taxonomy with human annotations.

### Step 1: Literature Review

The literature was reviewed to determine the initial topic taxonomy. We used 5 investigators to search the MEDLINE, EBSCO, and Web of Science databases with keywords “physician review topic,” “physician review,” “doctor review,” “topic taxonomy,” and “topic dimension” between 2013 and 2016. A total of 65 papers were found. Then, each investigator scored 65 papers on the relevance, 5 being the robust score and 1 being the weakest score. In addition, a paper was considered relevant if it included a taxonomy developed from the physician reviews. The agreement between any two investigators on the relevance score ranged from 0.6 to 0.81. Finally, we ranked the papers based on the score. After scoring each paper, we identified 7 relevant papers whose scores were >20 [20-26]. The 7 papers were carefully reviewed to identify the potential topics. Table 2 summarizes the topics identified (topics with different names but the same meaning were combined). The topics listed in Table 2 provided a good starting point to build the final topic taxonomy and helped to interpret the output of the LDA algorithm in step 2. As will be discussed later, the identified topics might be classified into 3 domains or high-level categories, including physician-related categories, patient-related categories, and system-related categories.

### Step 2: Data-Driven Analysis

The data-driven approach uses the LDA algorithm to explore the hidden topics among physician reviews. The approach consists of the following 2 steps.

#### Text Preprocessing

The preprocessing consists of several steps such as word segmentation, part-of-speech tagging, word stopping, and word replacement. Because the physician reviews are downloaded from a Chinese website and the Chinese words are not delimited, word segmentation is a necessary step. In this study, HanLP [27] was used to segment the Chinese text into a vector of words. HanLP provides part-of-speech tagging for each output token. Only meaningful phrases, such as nouns, verbs, adjectives, and adverbs, are retained after word segmentation. Therefore, the whole sentence is transformed into a vector with meaningful phrases.

**Table 2:** Physician review topics identified from the literature with related descriptions and references.

Topics	Description and reference
Physician knowledge and skill	Physician knowledge [20-23] Professional competence [21-23] Satisfaction with treatment [24]
Medical ethics (relational conduct)	Friendliness and caring attitude [24] Interpersonal style [21-23] Punctuality [20,25] Time spent with the patient [24]
Medicine and advice	Information and advice [24] Medicine and Pain control [26]
Communication	Communication attributes [22,24] Communication with patients [25] Communication with doctors [26]
Environment	Environment [26] Condition and equipment of a doctor’s office [25]
Business process	Appointment [25] Availability and accessibility [22] Other Staff [20,26] Responsiveness [26] Systems issues [21,23,26]
Financing	Cost of medical advice [22] Financing [26]

Another important step in preprocessing is word replacement. There are two reasons for word replacement. First, a synonym, a word having the same or nearly the same meaning as another word. Synonyms make the meaning more difficult to capture because the same meaning might have different forms. Synonyms are identified with the help of a synonym thesaurus (Harbin Institute of Technology IR-Lab Tongyici Cilin [28]). Second, to increase the number of instances for some named entities, such as body temperature, age, number, height, weight, and so on. For example, both 39°C and 40°C may refer to the body temperature. Therefore, we replaced them with the same special symbol, such as #BODY_TEMPERATURE#, to increase the number of cases for body temperature. Furthermore, a rule-based name entity recognition task was performed to recognize the important entities of the MUC-7 (Message Understanding Conference-7) framework, such as date, location, money, organization, percentage, person, and time [29].

#### Explorative Topic Discovery

Topic modeling was used to explore the hidden structure of physician review texts. Topic modeling is a type of statistical model for discovering the abstract “topics” that occur in a collection of documents. In this study, LDA was used as the tool for topic discovery [30,31]. LDA is a generative statistical model that allows sets of observations to be explained by unobserved categories that explain why some parts of the data are similar; for example, if observations are words collected into documents, LDA posits that each document is a mixture of a small number of topics and that each word’s creation is attributable to one of the document’s topics. In LDA, each document may be viewed as a mixture of various topics, where each document is considered to have a set of topics that are assigned to it via LDA. Moreover, a piece of text is generated as random mixtures over latent topics, where each topic is characterized by a distribution over words. LDA has been widely used to explore topics from the medical text [32-34].

**Figure 2:**
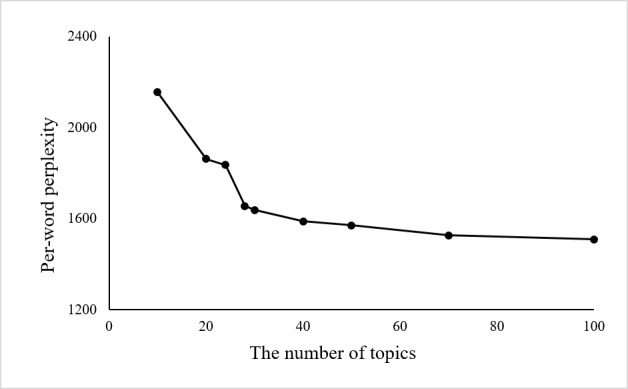
Per-word perplexity as a function of the number of topics.

An important question in LDA (and perhaps, for most cluster algorithms) is to determine the best number of categories (or clusters). In this study, the perplexity was used to determine the best category number. The perplexity measures the predictive power of competing models in language modeling. For a collection D of M reviews, the per-word perplexity is defined as: 
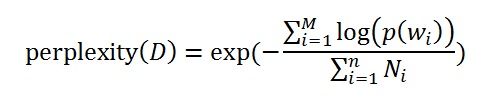


The perplexity can be understood as the predicted average number of equally likely words in certain positions, and it is a monotonically decreasing function of the log-likelihood [35,36]. Therefore, a lower perplexity over a held-out text means a higher log-likelihood, that is, better predictive performance. Figure 2 presents the predictive power of the LDA model in terms of the per-word perplexity for different numbers of topics.

As shown in Figure 2, the perplexity monotonically decreases with an increase in the number of topics, eventually tending to converge to a fixed value. Therefore, a higher number of topics is more preferred than a lower number. However, LDA often learns some topics that are difficult to interpret, and the model provides no tools for tuning the generated topics to suit an end-use application, even when time and resources exist to provide document labels. It is very difficult to interpret the medical or managerial meaning if the topic number is too large. Thus, there should be a balance between the perplexity and the interpretability. In this study, we chose the number of topics to be 30 because we observed that the perplexity decreases much more slowly when the topic number is >30. Table 3 presents the results of 30 identified topics; each topic is represented by a group of keywords. In addition, Table 3 provides the interpretation for each topic.

### Step 3: Human Annotation

After the data-driven approach for topic discovery, a human annotation was performed to finalize the topic taxonomy [37,38]. The topics identified from the literature (shown in Table 2) and the data (shown in Table 3) provided an initial topic taxonomy. We independently employed 10 graduate students who majored in information systems as coders to annotate 500 reviews each. They were asked to try to classify each review into the topics identified either in Table 2 or Table 3. Before the annotation started, they were asked to read another 200 reviews to familiarize themselves with the text. In addition, a training session was introduced to make sure each coder understood the meanings of the topics. When they encountered a review that could not be included in any previously identified topic, a new topic was created [39]. In this phase, the patient profile (PP) was created. Because each review was coded by 3 annotators, the discrepancies among the 3 coders were discussed until all conflicts were resolved. The agreement between any 2 coders on their initial review ranged from 0.6 to 0.81. After human annotation, some topics with similar content but different names (eg, topics 22 and 28 in Table 3) were combined. Table 4 presents the final topic taxonomy; it is a hierarchical taxonomy consisting of 3 high-level categories, namely physician-related categories, system-related categories, and patient-related categories. In addition, the explanations, keywords, and examples for each subtopic are provided.

### Automatic Topic Classification Algorithm

In this study, labeled-LDA was used to classify review texts into topics identified in the previous section. Labeled-LDA is a generative model for multilabeled corpora [40]. As a natural extension of both LDA (by incorporating supervised learning methods) and multinomial naïve Bayes (by incorporating a mixture model), it performs well in solving the problem of topic identification in multilabeled texts with improved the interpretability over LDA. The competitive advantage of labeled-LDA over a strong baseline discriminative classifier, such as a support vector machine, on multilabel text classification tasks has been validated by previous studies [40]. Multimedia Appendix 1 provides further details about labeled-LDA.

**Table 3:** Physician review topics discovered by the Latent Dirichlet Allocation.

Topic	Keywords	Interpretation
Topic 1	mood, confidence, from the heart, prospect, encourage, comfort, warm, relax, pressure, psychological distress, kind	Physician knowledge and skill (positive treatment evaluation)
Topic 2	state of an illness, patience, treatment, asking patients questions, carefully, detailed asking, serious and responsible, situation, interpretation, diagnosis, explain	Communication (asking and listening)
Topic 3	thanks, age, mother, daughter, health, son, sincerely, help, opportunity, once again, baby	Physician knowledge and skill (thanks for the positive results)
Topic 4	operate, success, test tube baby, transplant, eggs, failure, artificial insemination, thanks, natural, embryo, give up	Therapeutic schedule
Topic 5	thanks, work, a good man, grateful to, happiness, smooth life, health, express, family, wish, blessing	Physician knowledge and skill (thanks and positive results)
Topic 6	treatment, effect, symptoms, disease, obvious, turn for the better, combined therapy of Chinese and Western medicine, cure, ease one’s pain, acupuncture	Physician knowledge and skill (treatment effect)
Topic 7	at that time, in the heart, tell, really, no, worry about, know, be afraid of, nervous, feeling, happy	Communication (asking and listening)
Topic 8	good, no, online, really, more, looking for, give it a try, want, evaluation, have a try, experts	Business process (make an appointment)
Topic 9	see a doctor, successful, methods, treatment, hyperthyroidism, normal, indicators, drug, test, take the medicine, advice	Therapeutic schedule
Topic 10	good, attitude, better, special, patience, medical skill, kind, satisfaction, curative effect, technology, rare	Medical ethics (Relational conduct)
Topic 11	patient, time, experts, compare, need to do, experience, feeling, think, go to a doctor, trust, choose	Business process (make an appointment)
Topic 12	Traditional Chinese Medicine, toning your body, medicine, effect, Western medicine, proprietary Chinese medicine, drink, how long, adhere to, body	Medicine
Topic 13	check, operation, B ultrasound, report, inspection result, problem, reexamination, advice, test, draw blood, requirements	Medical examination
Topic 14	pregnancy, age, conceive, no, get married, treatment, infertility, friend, thank you, check, sperm	Disease symptoms (eg, infertility)
Topic 15	see a doctor, every time, time, patient, looking for, good, body, every day, disease, often, pay attention to	Medical ethics
Topic 16	surgery, in the hospital, out of the hospital, restore, thank you, treatment, arrange, excision, time, team, admitted to hospital	Business process (responsiveness, ward management)
Topic 17	patients, patience, problem, consulting, explain, thank you, touch, outpatient service, reply, online, encounter	Communication (explaining)
Topic 18	surgery, laparoscopic, the fallopian tubes, uterus, imaging, hysteroscopy, lining, found, uterine fibroids, adhesion, cyst	Therapeutic schedule
Topic 19	looking for, see a doctor, better, disease, attitude, good, introduce, friend, effect, cure	Physician knowledge and skill (treatment effect)
Topic 20	menstruation, pregnancy, follicles, ovulation, normal, monitoring, abortion, ovary, hormone, stimulate ovulation	Disease symptoms (eg, infertility)
Topic 21	hope, good, believe, really, feeling, confidence, better, best, think, as soon as possible, must be	Physician knowledge and skill (treatment effect, confidence)
Topic 22	cough, pneumonia, catch a cold, have a fever, how long, infusion, medicine, take the medicine, age, take an injection, turn for the better	Disease diagnosis (eg, infantile pneumonia and influenza)
Topic 23	medicine, prescribing, take the medicine, no, expensive, effect, cheap, capsule, disorderly, prescribe medicine disorderly, test	Medicine and prescribing
Topic 24	ask questions, do not, no, problem, impatient, want, speak, directly, medical records, to see a doctor, why	Communication (listening and explaining)
Topic 25	medical skill, medical ethics, noble, patients, good, exquisite, technology, worth, amiable, trust, enthusiasm	Physician skill and medical ethic
Topic 26	patient, attitude, seriously, patience, responsible, better, kindly, to see a doctor, careful, work, good	Medical ethics (Relational conduct)
Topic 27	do not, know, want, think, bad, why, problem, do not know, compare, comfortable, what do I do	Communication (listening and explaining)
Topic 28	treatment, diabetes, blood sugar, control, drug, insulin, diet, how long, stable, the state of illness, adjust	Disease diagnosis (eg, diabetes)
Topic 29	registered, time, line up, make an appointment, outpatient service, a plus sign, time, to see a doctor, difficult, particular requirement, expert registered ticket	Business process (make an appointment)
Topic 30	do not, good, no, how long, pain, serious, at a time, diarrhea, blood, already, appear	Physician knowledge and skill (pain control)

The corpus annotated in the last section was used to provide supervised information for labeled-LDA. The output was the word distribution for each topic as well as the topic distribution for each physician review. Multimedia Appendix 2 shows the top words learned by labeled-LDA (the Chinese words were translated into English). Each topic was illustrated using a word cloud map, in which the font size was proportional to the probability of the word occurring in the topic.

### Algorithm Evaluation

An evaluation was performed to estimate the performance of the proposed algorithm. The blind test bed consisted of 200 reviews covering all 9 diseases mentioned above. The algorithm output was compared with the human annotation results. Because the output of labeled-LDA was a distribution over all topics, we decided to label a topic only when its probability was higher than 1/L (*L* is the number of topics).

Table 5 presents the evaluation results. Precision, recall, and *F*-measure were used as the evaluation metrics [41]. Precision answers the following question:

Given all retrieved responses, what is the probability that the retrieved responses are relevant?

Recall answers the following question:

Given all relevant responses, what is the probability that the relevant responses are retrieved?

For classification tasks, the terms true positives (*tp*), true negatives (*tn*), false positives (*fp*), and false negatives (*fn*) compare the results of the classifier under test with trusted external judgments. The terms positive and negative refer to the classifier’s prediction (sometimes known as the expectation), and the terms true and false refer to whether that prediction corresponds to the external judgment (sometimes known as the observation). Precision and recall are then defined as: 
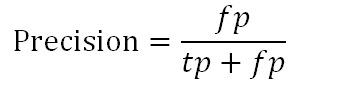

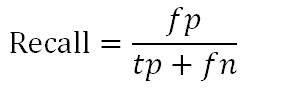


Because precision and recall are inversely related, the *F*-measure (also known as *F*-score) was used to evaluate the trade-off between them. In this case, an unweighted *F*-score was used. The unweighted *F-* score is the harmonic mean of precision and recall and is defined as: 
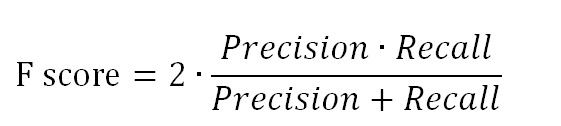


As shown in Table 5, the algorithm achieved impressive results with an average *F*-measure of 0.816 and with the highest score for hypertension (0.904) and the lowest score for infertility (0.682).

**Table 4:** The taxonomy of Web-based physician reviews (final).

Topic				
**Physician-related topics**			
		**Subtopic: Medical ethics**		
		Explanation	Physician’s interpersonal manners and behaviors perceived in the patient-physician interaction, including politeness, decency, patient participation in the treatment process, caring, listened to, understood, taken seriously.
		Keywords	Kind, good, attitude, medical ethics, patient, considerate, noble, careful, polite, show a patient every consideration, respect, indifference, pressing, unfriendly
		Examples	1. This doctor always gets to the treatment room on time and treats me with good manners. 2. At one point, our family member had inappropriate bleeding from several sites on the body which (a doctor) dismissed as unimportant. Eventually we were able to convince other people in the intensive care unit to take care of the situation.
		Original topic	LDA^a^ topic: 10, 15, 25, 26 Topic from the literature: Medical ethics (Relational conduct)
		Frequency (n)	2592
	**Subtopic: Medical competence**		
		Explanation	Patient’s perceptions of the doctor’s competence, experience and knowledge, including accuracy of the diagnostic process and treatment, safe practices or outcomes, observations of missed or correct care, and pain control.
		Keywords	Good, medical skill, curative effect, turn for the better, improved significantly, high degree of medical skill, recovered, exacerbation, aggravation
		Examples	1. His medical skill is so superb that I was soon discharged from the hospital. 2. Doctor Lin is very knowledgeable. He provided me with alternative treatment plans.
		Original topic	LDA topic: 1, 3, 5, 6, 19, 21, 25, 30 Topic from the literature: Physician knowledge and skill
		Frequency (n)	2391
	**Subtopic: Medical advice and prescription**		
		Explanation	The treatment solution given by a physician. It also includes the side effects of medications and treatments.
		Keywords	Treatment, drug, antibiotics, surgery, insulin, take the medicine, injection, oral drugs, infusion, azithromycin, chemotherapy
		Examples	1. Doctor Wang gave Gemcitabine, 5-fluorouracil for the treatment and explained it in detail in view of the different symptoms. 2. I did not get fat. My body shape is normal. No other side effects.
		Original topic	LDA topic: 4, 9, 12, 18, 23 Topic from the literature: Medicine and advice
		Frequency (n)	1910
	**Subtopic: Communication skills**		
		Explanation	Communication skills such as listening skills, asking skills and explaining skills. Make sure the patient is understood and address each patient’s question well.
		Keywords	Patiently answering question, explicate, cannot understand, do not get it, state of illness, problem, ask, detailed, explain, urge again and again, enjoin, analyze
		Examples	What doctor Wang said was easily understood. He will patiently explain what you do not understand.
		Original topic	LDA topic: 2, 7, 17, 24, 27 Topic from the literature: Communication
		Frequency (n)	2133
**System-related topics**			
	**Subtopic: Financing**		
		Explanation	The price for similar services, confusion about billing issues, the stress of dealing with billing departments and unexpected out-of-pocket costs.
		Keywords	Expenses, money, expensive, spend money, cheap, registration fee, waste money, inexpensive, difference, price, expenses for medicine, affordable price
		Examples	The service is good, but it’s expensive. Your insurance may not cover most of it. [...] Call the hospital and ask for the cost first before you commit to any specialty sessions [...]
		Original topic	Topic from the literature: Financing
		Frequency (n)	1903
	**Subtopic: Operation process**		
		Explanation	Discharge information, responsiveness, clinical environment and equipment, and make an appointment. Discharge information allows patients to report their feeling and experiences related to discharge, such as perceived diagnostic errors and revisits to the emergency department. Responsiveness paid attention to whether the physician was accessible when needed or not, such as when seeking emergency department care or immediate care, when the primary doctor is unavailable. Clinical environment and equipment focus on patients’ perception of the comfort of the environment of the hospital, quality and scarcity of equipment, and efficiency.
		Keywords	Difficult, make an appointment, see a doctor, flow, service, ward, consulting, wait in line, beds, reexamination, need to be, out of the hospital, online consulting
		Examples	After I got out of the hospital, doctor Liu called me several times. I told him my recent conditions. It took a long time before the ambulance reached the hospital. Fortunately, the doctors rescued the little boy from the jaws of death. I was very comfortable in the emergency room because at least one expert was on duty every night who regularly checked the patients. The doctor’s office was clean, and the hospital was not crowded. I was comfortable.
		Original Topic	LDA topic: 8, 11, 13, 16, 29 Topic from the literature: Environment, Business process
		Frequency (n)	1934
**Patient-related topics**			
	**Subtopic: Patient profile**		
		Explanation	Patient’s demographic information, including age, sex, address, occupation, diet, hobbies, etc
		Keywords	Gender, height, age, born, weight, address, habits, allergic, hair
		Examples	My aunt feels uncomfortable. She is 60 years old.
		Original Topic	Human annotation
		Frequency (n)	2339
	**Subtopic: Symptom**		
		Explanation	Symptom changes and sign changes. Symptom changes refer to subjective discomfort, abnormal feelings, and obvious pathological changes. Sign changes refer to the anomalous changes which could be diagnosed with objective tools.
		Keywords	Cough, symptoms, transfer, have a fever, abnormal, virus, high blood pressure, diarrhea, serious, catch a cold, thin, swelling, lymph node, bloodshot
		Examples	I have flu-like symptoms, for example, headache, cough, fever and rhinorrhea.
		Original topic	LDA topic: 14, 20
		Frequency (n)	1606
	**Subtopic: Diagnosis and pathogenesis**		
		Explanation	Disease types, features and pathogenesis.
		Keywords	Pathogenesis, influencing factor, pneumonia, diabetes, blood pressure, fallopian tubes, function, cause, infertility, congenital, gastric cancer, genetic abnormality
		Examples	I added a side dish of vegetables for my son. Soon he developed severe diarrhea, 10 times a day.
		Original topic	LDA topic: 22, 28
		Frequency (n)	1666

^a^LDA: Latent Dirichlet Allocation.

**Table 5:** The topic classification performance.

Disease	Precision	Recall	*F*-score
Infertility	0.672	0.738	0.682
Liver cancer	0.942	0.815	0.862
Influenza	0.742	0.795	0.762
Hypertension	0.863	0.958	0.904
Hyperthyroidism	0.947	0.774	0.821
Diabetes	0.883	0.878	0.871
Gastric cancer	0.927	0.704	0.795
Infantile pneumonia	1.000	0.722	0.820
Infantile diarrhea	0.967	0.745	0.826
Average	0.882	0.792	0.816

## Results

### Patient Listing Analysis Framework

Listening to patients is very important for health care providers to understand their customer needs and increase their satisfaction. The major results of patients’ interesting mining are summarized below. Welch’s *t* tests were conducted to test significant differences recorded on the topics for different groups of patients [42]. The *t* test can be used to determine if two datasets are significantly different from each other. Here is an example to illustrate the procedure for testing a given topic’s (eg, medical ethics [ME]) ratio differences for patients with acute diseases versus chronic diseases. First, we obtained the topic ratio for each message for topic ME from labeled-LDA algorithm’s output. For example, “message 1=0.245” indicates that message 1 contains 24.5% of the ME topic. To reduce unnecessary noise, topic ratios below 1/9 were set to 0. Then, we calculated relevant variables, such as the mean, SD, and sample size, for each group (ie, acute diseases vs chronic diseases), which were further used as inputs for the *t* test to determine whether there is a marked difference in the topic ratio for acute diseases versus chronic diseases.

### Acute Versus Chronic Diseases

**Figure 3:**
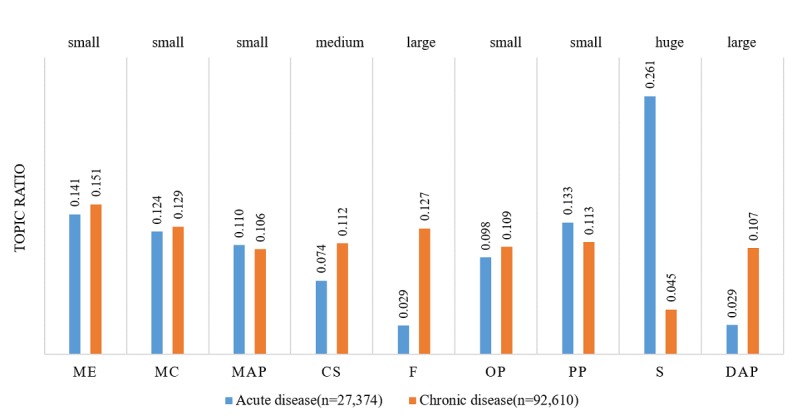
Topic ratio for acute and chronic diseases.Small (< 0.01), medium (0.01 - 0.20), large (0.20 - 0.50), very large (0.50 - 0.80) and huge (0.80 - 2.0) are effect sizes according to the magnitudes of Cohen's d. ME: Medical ethics; MC: Medical competence; MAP: Medical advice and prescription; CS: Communication skills; F: Financing; OP: Operation process; PP: Patient profile; S: Symptoms; DAP: Diagnosis and pathogenesis.

Influenza, infantile pneumonia, infantile diarrhea, and hyperthyroidism were categorized as acute diseases, whereas hypertension, diabetes, and infertility were categorized as chronic diseases. Figure 3 shows the interests of patients with acute and chronic diseases; the length of the bar represents the percentage of messages that contain a given topic; for example, a value of 0.141 for acute disease with topic ME means that 14.1% (14.1/100) of the messages for the acute disease group contained topic ME. The effect size (eg, small, medium, large, very large, and huge) was labeled for each topic to indicate the magnitude of the difference. The effect size was first measured by Cohen *d* [43] and then interpreted as small (<0.01), medium (0.01-0.20), large (0.20-0.50), very large (0.50-0.80), and huge (0.80-2.0) according to the values of Cohen *d* [44].

As shown in Figure 3, patients with acute diseases were more interested in symptoms (Cohen *d*=1.58, ∆*u*=0.216, *t*=229.75, and *P*<.001). Meanwhile, patients with chronic diseases were more interested in communication skills (CS; Cohen *d*=−0.29, ∆*u*=−0.038, *t*=−42.01, and *P*<.001), financing (F; Cohen *d*=−0.68, ∆*u*=−0.098, *t*=−99.26, and *P*<.001), and diagnosis and pathogenesis (DAP; Cohen *d*=−0.55, ∆*u*=−0.078, *t*=−80.09, and *P*<.001).

Patients with acute diseases were more interested in symptoms likely because symptoms are the most important factor for a patient describing an illness experience or during a consultation. In contrast, patients with chronic diseases were more concerned with CS, financing, and DAP. Because chronic diseases cannot be quickly cured and usually have an extended duration, patients are very familiar with their treatments and prescriptions. Therefore, medical competence (MC) and medical advice and prescription (MAP) are not the focus of their reviews. However, self-management and financial burden are major concerns for patients with chronic disease. These findings indicated that patients with different disease development rates (acute vs chronic) indeed exhibited different concerns to be addressed. Therefore, the training focus for physicians should be tailored to accommodate these distinct needs.

### Mild Versus Serious Diseases

**Figure 4:**
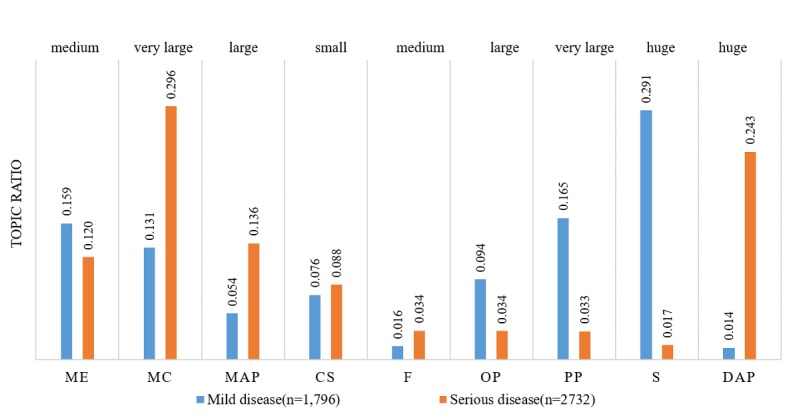
Topic ratio for mild and serious diseases. ME: Medical ethics; MC: Medical competence; MAP: Medical advice and prescription; CS: Communication skills; F: Financing; OP: Operation process; PP: Patient profile; S: Symptoms; DAP: Diagnosis and pathogenesis.

Influenza is categorized as a mild disease, whereas liver cancer and gastric cancer are categorized as serious diseases. Figure 4 shows the interests of patients with mild and serious diseases. Patients with mild diseases were more interested in ME (Cohen *d*=0.25, ∆*u*=0.039, *t*=8.33, and *P*<.001), operation process (OP; Cohen *d*=0.57, ∆*u*=0.060, *t*=18.75, and *P*<.001), PP (Cohen *d*=1.19, ∆*u*=0.132, *t*=39.33, and *P*<.001), and symptoms (S; Cohen *d*=1.91, ∆*u*=0.274, *t*=62.82, and *P*<.001). Patients with serious diseases were more interested in MC (Cohen *d*=−0.99, ∆*u*=−0.165, *t*=−32.58, and *P*<.001), MAP (Cohen *d*=−0.65, ∆*u*=−0.082, *t*=−21.45, and *P*<.001), financing (F; Cohen *d*=−0.26, ∆*u*=−0.018, *t*=−8.45, and *P*<.001), and DAP (Cohen *d*=−1.55, ∆*u*=−0.229, *t*=−50.93, and *P*<.001).

Patients with mild disease were more interested in ME, OP, PP, and symptoms because mild diseases are usually simple, nonlife-threatening, and easy to cure. Therefore, these patients placed more attention on the service quality and patient empowerment. In contrast, we observed that patients with serious diseases were more concerned with MC, MAP, financing, and DAP. Because serious diseases are usually complicated, life-threatening, and carry a heavy financial burden, the disease pathogenesis, treatment method, and financial issues are the major concerns for these patients. These findings demonstrated that patients presenting with different disease severities exhibited different interests or concerns to be addressed.

### High- Versus Low-Level Hospitals

**Figure 5:**
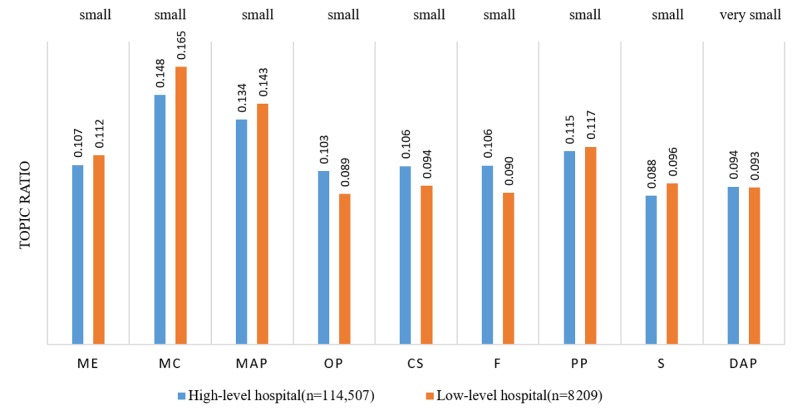
Topic ratio for high level and low level hospitals. ME: Medical ethics; MC: Medical competence; MAP: Medical advice and prescription; CS: Communication skills; F: Financing; OP: Operation process; PP: Patient profile; S: Symptoms; DAP: Diagnosis and pathogenesis.

A-level hospitals were categorized as high-level hospitals, whereas B- and C-level hospitals were categorized as low-level hospitals. The topic ratios were compared with the hospital level, as seen in Figure 5. Patients at high-level hospitals were more interested in OP (Cohen *d*=0.08, ∆*u*=0.014, *t*=6.73, and *P*<.001), CS (Cohen *d*=0.08, ∆*u*=0.012, *t*=8.33, and *P*<.001), and financing (F; Cohen *d*=0.10, ∆*u*=0.016, *t*=7.07, and *P*<.001).

 However, the effect sizes for those differences were small. Patients at low-level hospitals were more interested in ME (Cohen *d*=−0.09, ∆*u*=−0.005, *t*=−7.58, and *P*<.001), MC (Cohen *d*=−0.05, ∆*u*=−0.017, *t*=−4.48, and *P*<.001), MAP (Cohen *d*=−0.03, ∆*u*=−0.009, *t*=−2.88, and *P*<.01), and symptoms (S; Cohen *d*=−0.04, ∆*u*=−0.008, *t*=−3.67, and *P*<.001); however, the effect sizes for those differences were also small.

It is clear that customer demand in high-level hospitals likely differed from that in low-level hospitals. As shown in Figure 5, ME and MC are the two most outstanding concerns for patients from low-level hospitals. In addition, MC is a concern because physician competence at low-level hospitals might be reduced compared with high-level hospitals, thus worrying patients. ME is another concern because customers of low-level hospitals are mostly low-income patients with little education, making conflicts with doctors or nurses more likely. Patient distrust in low-level hospitals, such as small clinics, village health clinics, community health service stations, and neighborhood health centers, in China has been reported in several prior studies [45]. Multimedia Appendix 3 describes complete comparisons of topic ratio differences across the disease type and hospital level.

## Discussion

### Principal Findings

In this study, we build a taxonomy that includes 3 domains or high-level categories and 9 subtopics or low-level categories using mixed methods. Then, a classification algorithm based on labeled-LDA is proposed. The evaluation result shows that the *F*-measure of the proposed classification algorithm reaches 0.816 on average. Our analysis on large review corpus suggests that patients with different disease types or hospital levels have different concerns to be addressed.

### Comparison With Prior Work on the Taxonomy Development Method

Prior work on developing a physician review taxonomy mainly follows one of the three approaches. The first approach is a literature review [22]; for example, Boquiren et al [22] reviewed empirical studies that were published from 2000 to November 2013 to determine the primary domains underlying the patient satisfaction with the doctor. However, it is very hard to identify any new dimension by summarizing the literature, which is a major challenge for our fast-changing environment. The second approach is content analysis [24-26]; for example, Emmert et al [24] did a content analysis of 3000 randomly selected narrative comments from a German PRW and developed a theoretical categorization framework addressing physician-, staff-, and practice-related patient concerns. However, the manual nature of the content analysis makes it very hard to process large amounts of reviews, which is another challenge of the information age. The third approach is the algorithm or data-driven approach [9,20,21]; for example, Hao and Zhang [9] applied the topic extraction algorithm LDA to >500,000 textual reviews from >75,000 Chinese doctors across 4 major specialty areas to identify the dimensions inside the physician reviews. However, the output of the algorithm usually depends on the data, and many categories are hard to explain in medical practice. Some studies from other domains have also used the algorithm approach; for example, Guo et al [42] identified the key dimensions of customer service voiced by hotel visitors with a data mining approach LDA; they uncovered 19 controllable key dimensions that are important for managing hotels’ interactions with visitors.

The three approaches have both advantages and disadvantages. In this study, we propose a new method that combines the three approaches mentioned above. We start with the literature review approach to form an initial taxonomy. Then, an algorithm-based data-driven approach is used to explore and find more categories. Finally, a human annotation approach is used to finalize the taxonomy. This study demonstrates the plausibility and validity of the proposed mixed method.

### Comparison With Prior Work on Patients’ Interests Mined from Web-Based Reviews

Some researchers have manually coded and identified patients’ interests from the Web-based reviews; for example, López et al [38] summarized two topic categories as global themes (which included the overall excellence, recommendation, negative sentiment, intent not to return, and professionalism) and specific factors (including interpersonal manner, technical competence, and system issues). Espinel et al [23] built a taxonomy for physician comments through an analysis of Web-based physician reviews, physician-related and system-related. In detail, physician-related topics included 2 subtopics, interpersonal style and technical skills and knowledge and preparation, whereas system-related topics included scheduling, wait time, parking, location, and cleanliness. Boquiren et al [22] revealed 5 broad domains underlying the patient satisfaction with the doctor—communication attributes, relational conduct, technical skill and knowledge, personal qualities, and availability and accessibility. Tymiński et al [25] identified patients’ criteria for assessment of doctors—kindness and propriety, punctuality, communication with patients, condition and equipment of a doctor’s office, length of the appointment, and cost of the medical advice. Davis and Hanauer [46] identified the key themes associated with positive and negative patient reviews. Themes that emerged from the high- and low-scoring reviews were similar in content but opposite in valence. Notably, physician-specific themes included temperament, knowledge and competency, physical examination, communication abilities, and mindfulness of cost. Practice-specific themes included scheduling, staff temperament, office cleanliness, waiting room, and insurance.

Some other researchers have applied the algorithm to extract patients’ interests from the Web-based reviews automatically; for example, Hao and Zhang [9] applied LDA to >500,000 textual reviews of >75,000 Chinese doctors across 4 major specialty areas. They found the following important topics from the reviews: treatment effects, technical skills, appreciate the surgery result, story of treatment, story of surgery, bedside manner, story of registration, story of finding doctors, general appreciation, description of symptoms, and concern about children’s health. In addition, Wallace et al [21] analyzed a corpus comprising nearly 60,000 such reviews with a state-of-the-art probabilistic model of text factorial LDA; they suggested three important topics of patients’ interests as systems, technical, and interpersonal. There are also researchers who identified users’ interests from Web-based health communities; for example, Lu et al [47] developed a new content analysis method using text mining techniques to determine hot topics of concern. They identified 5 significantly different health-related topics: symptom, examination, drug, procedure, and complication.

### Conclusions

This study explores the internal dimensions of Web-based physician reviews, proposes an automatic classification algorithm based on labeled-LDA, and uses patient listening as an application to illustration the value of physician review mining. The identified taxonomy includes three high-level domains or categories and many subcategories or subtopics. The evaluation of the result of the proposed classification algorithm achieved impressive results with an *F*-measure of 0.816 on average with the highest *F*-measure for hypertension (0.904) and the lowest for infertility (0.682). The mining results indicate that symptoms are more often mentioned by patients with acute diseases, whereas CS, financing, and DAP are more often mentioned by patients with chronic diseases. Patients with mild diseases are more interested in ME, OP, PP, and symptoms. Meanwhile, patients with serious diseases are more interested in MC, MAP, financing, and DAP.

This study has some practical implications. First, this study provides an efficient and cost-effective way to analyze large amounts of physician reviews automatically. With the popularity of PRWs, the information overload has become a major concern for users. The taxonomy and method proposed in this study provide a convenient way to listen to patients, which is a very important step toward patient-centered care. In addition, the illustrated findings from physician review mining indicate that patients with different diseases and from different hospital levels might have different concerns that need to be addressed. Second, the taxonomy and algorithm proposed in this study also provide the bases for building decision aid tools that help patients make better decisions regarding the physician choice; for example, the decision aid tools might describe a physician with prominent tags such as good communication and high competence. Furthermore, the system can visualize different dimensions of a physician in a graph or compare multiple physicians across different dimensions in a matrix.

Although the taxonomy developed in this study occurred within a Chinese context, it is still generalizable to other countries and languages for the following two reasons. First, the initial taxonomy that serves as the starting point for this study was derived from the literature of Western countries. Therefore, the final taxonomy should also have a strong connection with those countries or languages. Second, China is a very large country. The economy in the eastern district is prosperous, whereas the western district is far behind. The intracountry diversity of China is quite high, rivaling or exceeding the intercountry differences of some continents (eg, Europe). Therefore, we believe that China is a good example that reflects the medical needs of both developed and developing countries.

This research also has three limitations. First, this study is descriptive in nature. Therefore, the correlation between important variables (eg, satisfaction and topics) cannot be revealed in this study. Second, the algorithm proposed in this study relies more or less on counting the prevalence of words rather than evaluating them positively or negatively. Some parts of the reviews may only reflect a mere description of an encounter. Often, people describe an encounter and in the end, give an evaluation of some specific aspects of that encounter. If the algorithm only counts frequencies, it may not get to the bottom of the real review’s motivation in this respect. Third, the results of this study could be biased because of fake reviews because anyone visiting a physician can write a review on the website, and the review process is anonymous. Although fake reviews are a common limitation to Web-based review studies, a fake review detection algorithm (eg, Yelp’s fake review filter) can be applied in the future to increase study rigor.

Future research might include identifying hygienic and other motivating factors underlying for patient satisfaction. One avenue of research may be to explore a two-factor theory that states that certain factors cause user satisfaction, whereas a separate set of factors cause dissatisfaction. Because we can collect the user satisfaction data from PRWs, such an exploratory study is now practical in conjunction with additional sentiment analyses to determine a valence score for each dimension. The basic idea of the two-factor theory is that factors that lead to satisfaction or dissatisfaction are different. Some factors only relate to satisfaction, whereas others only relate to dissatisfaction. Understanding dissatisfying factors that demotivate and satisfying factors that motivate is important information for health care providers who want to improve user satisfaction in a cost-effective and patient-centered manner.

## Appendix

Multimedia Appendix 1 Details for Labeled LDA.

Multimedia Appendix 2 Word cloud visualization of topics (n=122,716).

Multimedia Appendix 3 The topic ratio differences across disease types, city levels and hospital levels.

## Abbreviations

CS: communication skills
DAP: diagnosis and pathogenesis
LDA: Latent Dirichlet Allocation
MAP: medical advice and prescription
MC: medical competence
ME: medical ethics
OP: operation process
PP: patient profile
PRW: physician review websites
RQ: research question
