# Supplementary material for: Developing Embedded Taxonomy and Mining Patients’ Interests From Web-Based Physician Reviews: Mixed-Methods Approach
Source: J Med Internet Res. 2018 Aug 16;20(8):e254. doi: 10.2196/jmir.8868 (PMC6117498; doi:10.2196/jmir.8868)
Supplement: Multimedia Appendix 1 [file jmir_v20i8e254_app1.pdf]

Details for Labeled LDA

Labeled LDA is a probabilistic graphical model that describes a process for generating a labeled document collection . Unlike LDA, Labeled LDA incorporates supervision by simply constraining the topic model to use only those topics that correspond to a document's (observed) label set. The graphical model of Labeled LDA is shown in Figure 1.

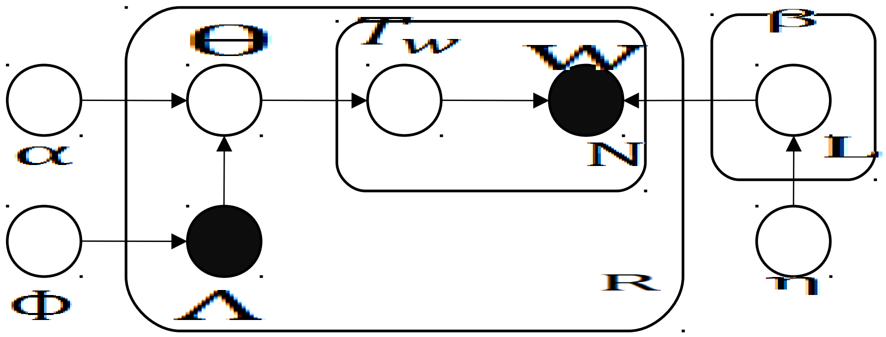

Figure 1 Graphical model of Labeled LDA

Each review is represented by a list of word $w^{(r)} = (w_1, \cdots, w_i, \cdots w_{N_r})$ and a list of topic label $\Lambda^{(r)} = (b_1, \cdots, b_j, \cdots b_L)$ where $w_i \in \{1, \cdots, K$ and $b_j \in \{0,1\}$. Here N_r is the word number of review r, L is the total number of topic label, and K is the size of vocabulary. The generative process consists of the following steps:

For each topic $l \in \{1, \cdots, L\}$:

Generate $\beta_l = (\beta_{l,1}, \cdots, \beta_{l,K})^T \sim Dir(\cdot\,|\eta)$

For each review r:

For each topic $l \in \{1, \cdots, L\}$:

Generate $\Lambda_l^{(r)} \in \{0,1\} \sim \text{Bernouli}(\cdot\,|\Phi_l)$

$\lambda^{(r)} = \{l | \Lambda_l^{(r)} = 1\}$

$M_{ij}^{(r)} = \begin{cases} 1 & \lambda_i^{(r)} = j \\ 0 & \text{otherwise} \end{cases}$ $d_r = |\lambda^{(r)}|,\ i \in \{1, \cdots, d_r\}, j \in \{1, \cdots, L\}$

Generate $\alpha^{(r)} = M^{(r)} \times \alpha = (\alpha_{\lambda_1^{(r)}}, \cdots, \alpha_{\lambda_{d_r}^{(r)}})^T$

Generate $\theta^{(r)} = (\theta_{l_1}, \cdots, \theta_{l_{d_r}})^T \sim Dir(\cdot\, |\alpha^{(r)})$

For each word w_i, i in $\{1, \cdots, N_r\}$:

Generate $T_{w_i} \in \{\lambda_1^{(r)}, \cdots, \lambda_{d_r}^{(r)}\} \sim Mult(\cdot\, |\theta^{(r)})$

Generate $\theta^{(r)} \in \{1, \cdots, K\} \sim Mult(\cdot\, |\beta_{T_{w_i}})$

Note: β_l is a vector parameter of multinomial distribution, $\alpha = (\alpha_1, \cdots, \alpha_l)^T$ is the prior of Direchlet topic distribution, η *is* the prior of Direchlet word distribution, Φ_l is the label prior of topic *l*, $M^{(r)}$ is a projection matrix.

In this paper, we use collapsed Gibbs sampling to train manual annotated samples . The sampling probability for a topic for w_i in review r in L-LDA is represented as:

$$\mathrm{P}\left(T_{w_i} = \mathrm{j} \middle| T_{-w_i}\right) \propto \frac{n_{-w_i,j}^{w_i} + \eta w_i}{n_{-w_i,j}^{(\cdot)} + \eta^T 1} \times \frac{n_{-w_i,j}^{(r)} + \alpha_j}{n_{-w_i,\cdot}^{(r)} + \alpha^T 1}$$

where $n_{-w_i,j}^{w_i}$ is the number of word w_i in topic $j \in \lambda^{(r)}$.

References

1. Ramage D, Hall D, Nallapati R, Manning CD, editors. Labeled LDA: A supervised topic model for credit attribution in multi-labeled corpora. Proceedings of the 2009

Conference on Empirical Methods in Natural Language Processing; 2009; Singapore: Association for Computational Linguistics.
2. Griffiths TL, Steyvers M. Finding scientific topics. Proceedings of the National Academy of Sciences of the United States of America. 2004;101 Suppl 1(1):5228.
