# Supplementary figures and images for: Developing Embedded Taxonomy and Mining Patients’ Interests From Web-Based Physician Reviews: Mixed-Methods Approach
Source: J Med Internet Res. 2018 Aug 16;20(8):e254. doi: 10.2196/jmir.8868 (PMC6117498; doi:10.2196/jmir.8868)

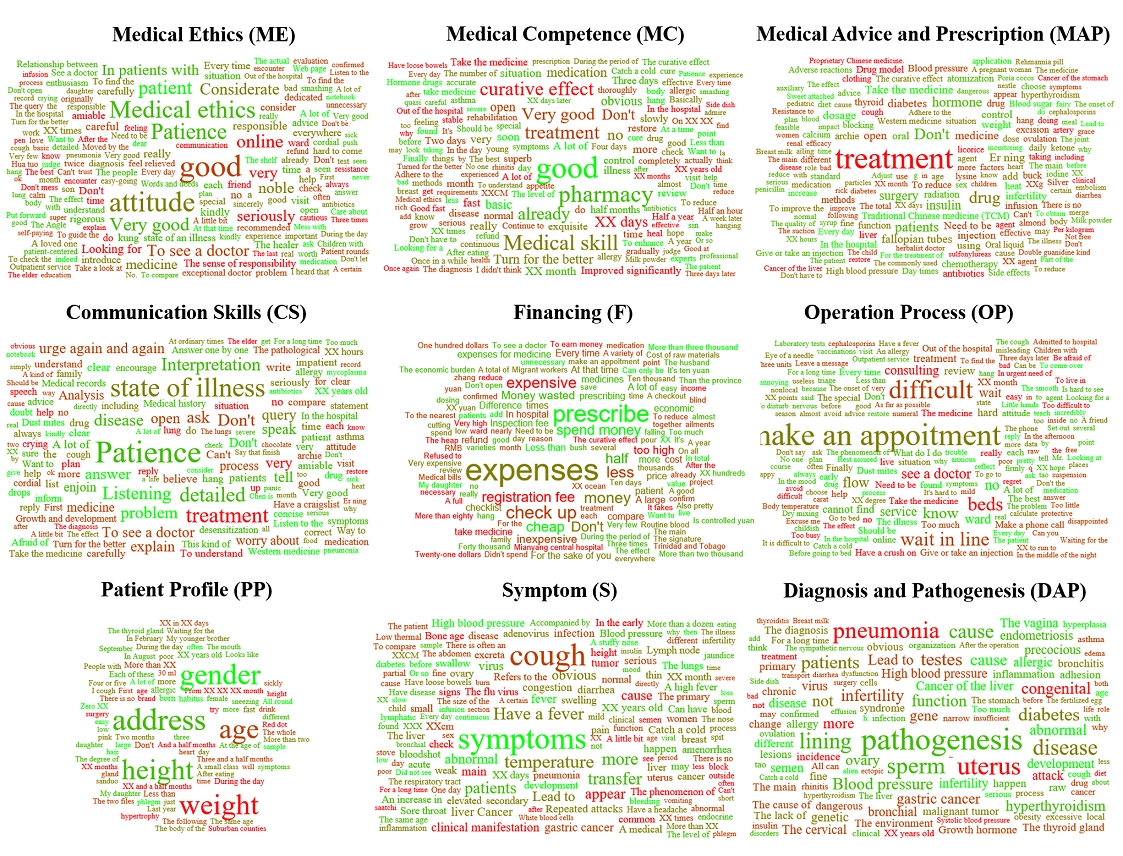

Supplement: Multimedia Appendix 2 [file jmir_v20i8e254_app2.png]
