# Supplementary material for: Developing Embedded Taxonomy and Mining Patients’ Interests From Web-Based Physician Reviews: Mixed-Methods Approach
Source: J Med Internet Res. 2018 Aug 16;20(8):e254. doi: 10.2196/jmir.8868 (PMC6117498; doi:10.2196/jmir.8868)
Supplement: Multimedia Appendix 3 [file jmir_v20i8e254_app3.pdf]

The topic ratio differences across disease types and hospital levels

Table 1 Topic ratio differences for acute and chronic diseases

Topic	Topic ratio of acute disease	Topic ratio of chronic disease	Cohen's d	Δu	t	P
ME	0.141	0.151	-0.06	-0.010	-8.46	.000***
MC	0.124	0.129	-0.03	-0.005	-4.67	.000***
MAP	0.110	0.106	0.03	0.004	3.77	.000***
CS	0.074	0.112	-0.29	-0.038	-42.01	.000***
F	0.029	0.127	-0.68	-0.098	-99.26	.000***
OP	0.098	0.109	-0.07	-0.011	-10.42	.000***
PP	0.133	0.113	0.14	0.020	20.94	.000***
S	0.261	0.045	1.58	0.216	229.75	.000***
DAP	0.029	0.107	-0.55	-0.078	-80.09	.000***

Note: *** P < .001

Table 2 Topic ratio differences for mild and serious diseases

Topic	Topic ratio of mild disease	Topic ratio of serious disease	Cohen's d	Δu	t	P
ME	0.159	0.120	0.25	0.039	8.33	.000***
MC	0.131	0.296	-0.99	-0.165	-32.58	.000***
MAP	0.054	0.136	-0.65	-0.082	-21.45	.000***
CS	0.076	0.088	-0.10	-0.012	-3.3	.010**
F	0.016	0.034	-0.26	-0.018	-8.45	.000***
OP	0.094	0.034	0.57	0.060	18.75	.000***
PP	0.165	0.033	1.19	0.132	39.33	.000***
S	0.291	0.017	1.91	0.274	62.82	.000***
DAP	0.014	0.243	-1.55	-0.229	-50.93	.000***

Note: ** P < .01, *** P < .001

Table 3 Topic ratio differences for high level and low level hospitals

Topic	Topic ratio of high level hospitals	Topic ratio of low level hospitals	Cohen's d	Δu	t	P
ME	0.107	0.112	-0.09	-0.005	-7.58	.000***
MC	0.148	0.165	-0.05	-0.017	-4.48	.000***
MAP	0.134	0.143	-0.03	-0.009	-2.88	.004**
CS	0.103	0.089	0.08	0.014	6.73	.000***
F	0.106	0.094	0.10	0.012	8.33	.000***

OP	0.106	0.090	0.08	0.016	7.07	.000***
PP	0.115	0.117	-0.02	-0.002	-1.69	.091
S	0.088	0.096	-0.04	-0.008	-3.67	.000***
DAP	0.094	0.093	0.00	0.001	0.1	0.917

Note: ** P < .01, *** P < .001
